# α-Asarone Attenuates Osteoclastogenesis and Prevents Against Oestrogen-Deficiency Induced Osteoporosis

**DOI:** 10.3389/fphar.2022.780590

**Published:** 2022-03-18

**Authors:** Hao Tian, Tao Jiang, Kai Yang, Ruonan Ning, Tianqi Wang, Qi Zhou, Niandong Qian, Ping Huang, Lei Guo, Min Jiang, Xiaobing Xi, Xing Xu, Lianfu Deng

**Affiliations:** ^1^ Department of Orthopaedics, Shanghai Key Laboratory for Prevention and Treatment of Bone and Joint Diseases, Shanghai Institute of Traumatology and Orthopaedics, Ruijin Hospital, Shanghai Jiao Tong University School of Medicine, Shanghai, China; ^2^ Department of Endocrine and Metabolic Diseases, Ruijin Hospital, Shanghai Jiao Tong University School of Medicine, Shanghai, China

**Keywords:** α-asarone (ASA), osteoclasts, osteoporosis (OP), bone resorption, bone loss

## Abstract

Osteoporosis (OP) is defined as low bone mineral density which features over activated osteoclasts (OCs) and bone resorption. Targeting excessive OCs activity is thought to be an effective therapeutic approach for OP treatment. α-asarone (ASA), a compound from the traditional Chinese medicinal herb *Acorus tatarinowii*, has been widely used as a therapeutic agent against several diseases such as epilepsy, cough, bronchitis and asthma for many years. Recently, it was reported that ASA-derived lignins which were purified from *Acorus tatarinowii* root tissues effectively suppressed both RANKL-induced osteoclastogenesis and bone resorption. Besides, a classic Chinese formulation Bajitianwan (BJTW) which consisted of root and rhizome of *Acorus tatarinowii Schott* also showed positive effects on age-related bone loss. In the present study, we aimed to study the effects of ASA on osteoclastogenesis *in vitro* and *in vivo.* As illustrated by TRAP staining, ASA was capable of inhibiting RANKL-induced osteoclastogenesis in a dose-dependent manner, not only at an early-stage, but also in the late-stage. Besides, it also effectively suppressed bone resorption of mature OCs in a pit resorption assay. The formation of F-actin ring during osteoclastogenesis, which was important in OCs bone-resorption, was impaired as well. Subsequent mechanism experiments exposed that ASA inhibited osteoclastogenesis related genes in a time-dependent manner through AKT, p38 and NF-κB, followed by NFATc1/c-fos signaling pathway. Notably, our *in vivo* study uncovered that ASA was capable of improving the bone microstructure in oestrogen-deficiency induced OP models. Thus, our current work highlighted the important role of an old drug ASA in bone metabolism especially in OCs differentiation. ASA may find its potential as a lead compound to treat excessive OCs activity-induced bone loss diseases and more structure optimization is further needed.

## Introduction

Bone is dynamically remodeled through lifetime by the synchronized action of osteoblasts (OBs) and osteoclasts (OCs), cells that deposit and reabsorb bone, respectively (Boyle et al., 2003; Indo et al., 2013; Furuya et al., 2018).

OCs are polynuclear giant cells with bone-resorbing capability, which derived from bone marrow-derived macrophage cells (BMMs) (Boyle et al., 2003; Asagiri and Takayanagi 2007; Indo et al., 2013). Skeletal stem cells generate OBs, the bone-forming cells, which produce new bone by secreting bone matrix and become entombed in the calcified matrix as osteocytes.

Disruption of the harmonious balance between OBs and OCs activities will cause various diseases, such as osteosclerosis promoted by hypo-activity of OCs and osteoporosis (OP) by hyper-activity of OCs. OP is a chronic, metabolic and systemic skeletal disorder characterized by deceased bone mineral density and impaired bone microarchitecture (Sözen et al., 2017), resulting in numerous clinical and health-related consequences, including osteoporotic fractures, vertebra collapses, the need for long-term care, and excess mortality (Compston et al., 2019).

According to the statistics from the International Osteoporosis Foundation in 2005, almost 200 million people were suffering from OP among all populations (Burge et al., 2007). One in three women over the age of 50 and one in five men will experience osteoporotic fractures in their lifetime, and it is predicted that osteoporotic fractures may increase by 49%, with annual costs projected to reach $25 billion by 2025 (Burge et al., 2007). Therefore, controlling OP is essential in lessening the personal and socioeconomic burden of osteoporotic fractures as well as other health-related consequences (Sözen et al., 2017; Compston et al., 2019).

Osteoclasts play a prominent role in bone homeostasis, together with osteoblasts and osteocytes. Macrophage colony-stimulating factor (M-CSF) (Yoshida et al., 1990) and receptor activators of the nuclear factor-κb (NF-κB) ligand (RANKL) (Kong et al., 1999; Xiong et al., 2018) are the most essential cytokines in OCs differentiation. M-CSF provides monocytes with survival, proliferation and differentiation signals during BMMs osteoclastogenesis, while RANKL provides OCs precursor cells with signals to differentiate into mature OCs. RANKL provides differentiation signals through binding to RANK on OC precursor cells (Suda et al., 1999; Aubin and Bonnelye 2000). RANKL/RANK initiates trimerization of RANK and recruits tumor necrosis factor receptor associated factor 6 (TRAF6) to trigger downstream osteoclastogenic signaling cascades, including ERK1/2, JNK 1/2, p38, NF-κB and AKT signal pathways. Then nuclear factor of activated T-cells, cytoplasmic 1 (NFATc1)/c-fos pathway, which has been revealed as the master pathway in regulating osteoclastogenesis, is activated (Ishida et al., 2002). Eventually, BMMs will differentiate into functionally mature OCs, forming a ruffled border with an isolated resorption microenvironment on the bone surface to resorb bone matrix. In this isolated resorption microenvironment, multiple acids and proteases such as tartrate-resistant acid phosphatase (TRAP) (van de Wijngaert and Burger 1986), Cathepsin K (CTSK) (Saftig et al., 1998), Matrix metalloproteinase 9 (MMP9) (Wucherpfennig et al., 1994) will be secreted to resorb bone matrix.

Because of the vital role of aberrant OCs differentiation and function in bone disorders, agents that suppress RANKL signaling pathway have been attracting the attention of researchers. Based upon the mechanism, anti-osteoclastogenesis therapies including nitrogen-containing bisphosphonates and RANKL monoclonal antibody, denosumab emerged and became conventional treatments for OP. However, bisphosphonates and denosumab potentially cause serious side effects, such as Medication-Related Osteonecrosis of the Jaw (MRONJ), which are called Denosumab-Related Osteonecrosis of the Jaws (DRONJ) (Egloff-Juras et al., 2018) and Bisphosphonate-Related Osteonecrosis of the Jaws (BRONJ) (Hewitt and Farah 2007), respectively, while the efficient therapy against MRONJ still remains controversial and international consensus on the best treatment strategy has not been established yet (Kawahara et al., 2021). Thus, finding new osteoclastgenesis modulators with a more effective and safer manner will facilitate the treatment for OP and prevention for fractures.

Despite the advances in anti-osteoclastogenesis agent discovery, high costs and poor productivity remain a challenge for new drug development. Drug repurposing that identifies novel applications for old drugs (including approved or investigational agents), is an efficient and preferred strategy to develop an entirely new agent for a given indication (Pushpakom et al., 2019). One of the most important advantages is that the old drugs have sufficient safety data in preclinical models and humans, which will decrease the risk of failure (Strittmatter 2014).

An old drug that has aroused considerable interest is α-asarone (trans-asarone), a naturally produced phenylpropene isolated from several plants, especially the Chinese medicinal herb *Acorus tatarinowii*. *Acorus tatarinowii* is a perennial herb clinically administered for the treatment of stroke, depression, seizure, mental disorders, dementia, rheumatosis and inflammatory diseases. Among the extracts from *Acorus tatarinowii*, propenylic asarones and asarone-containing extracts are in the focus of pharmacological interests, since it is proposed to exert multiple effects such as antidepressant, antihyperlipidemic, anticholestatic, anti-inflammatory, anticarcinogenic, anticonvulsive, antimicrobial, mucoidal, insecticidal, antioxidant, antithrombotic, anxiolytic, neuroprotective and radioprotective effects (Uebel et al., 2021). Among three propenylic asarones isoforms (including α-asarone, β-asarone and γ-asarone), α-asarone (ASA) is the safest one which has been used clinically to treat epilepsy, cough, bronchitis and asthma for many years, whereas the use of β-asarone is restricted and the toxicodynamic profile of γ-asarone remains unknown (Chellian et al., 2017; Uebel et al., 2021). Besides, ASA has aroused extensive research interest due to its various pharmacological activities, such as anxiolytic-like effects, improvement of learning, relief of memory disorders, anti-atherosclerosis effects and so on (Park et al., 2017; Zeng et al., 2021; Zhu et al., 2021).

Recently, ASA and ASA-derived lignins stimulate interest in the field of bone remodeling. A classic Chinese formulation Bajitianwan (BJTW) which consisted of root and rhizome of *Acorus tatarinowii Schott* exerted positive effects on age-related bone loss (Xu et al., 2020). Besides, ASA-derived lignins such as Tatarinan O, Tatarinan N, Tatarinan T purified from root tissues of *Acorus tatarinowii* were reported to effectively suppress both osteoclastogenesis and bone resorption induced by RANKL (Xu et al., 2016a; Zhang et al., 2018; Zhang et al., 2019).

Considering the effects of *Acorus tatarinowii*, ASA and ASA-derived lignins in multiple areas, we were elicited to explore the effects of ASA in bone metabolism. In the present study, we discovered that ASA deceased OCs differentiation. TRAP staining, F-actin staining, RT-PCR, Western Blotting were applied to investigate its effects and mechanisms in osteoclastogenesis *in vitro*. We also investigated the effects of ASA on OVX-induced osteoporotic mice. As shown in micro-computed tomography (micro-CT) and histomorphometry analysis, ASA improved bone microstructure *in vivo*. Our study will help to provide a potential option and a lead compound for the treatment of bone loss diseases.

## Materials and Methods

### Materials

α-asarone (ASA) was purchased from MedChemExpress. Dimethyl sulfoxide (DMSO), TRAP staining kit were purchased from Sigma-Aldrich. CCK8 kit was from Dojindo Molecular Technology. ɑ-modified eagle’s medium (ɑ-MEM), fetal bovine serum (FBS), penicillin and streptomycin (PS) were from Thermo Fisher Scientific. M-CSF and RANKL were obtained from Novoprotein Scientific Inc. TRIZOL was purchased from Invitrogen, reverse transcript reagents and SYBR Green PCR Master Mix were from Takara Biotechnology. Primers were synthesized from Invitrogen. Phalloidin-iFlour™ 555 Conjugate was from AAT bioquest. Inc. Antibodies for c-fos, NFATc1, JNK1/2, p-JNK1/2, ERK1/2, p-ERK1/2, p-p38, p38, p-p65, p65, p-IκB-α, AKT, p-AKT, CSTK, TRAP, MMP9 and β-actin were from Cell Signal Technology.

### Isolation and Culture of Bone Marrow-Derived Macrophage Cells and Osteoclasts

The procedure of obtaining BMMs for OCs differentiation was a standard method which was described previously (Xu et al., 2016b). To obtain BMMs, bone marrow cells were collected from the femur and tibiae of four-week-old male C57/BL6 mice. Briefly, the bone marrow cells in the femur and tibiae were flushed out and washed. After removing the red blood cells, cells were incubated in full medium containing ɑ-MEM, 10% FBS, 1% penicillin/streptomycin in an incubator at 37°C with 5% CO_2_. After 16 h, the supernatant cells were collected and maintained in full medium with 30 ng/mL M-CSF for another 2 days. Then the cells adhering to the bottom of the dish were classified as BMMs. BMMs were then harvested and cultured in full medium plus 30 ng/mL M-CSF and 50 ng/ml RANKL for additional 5-7 days to obtain mature OCs. Since cells obtained from different animals may respond differently, we mixed the cells from two different mice each time for BMM cultures to minimize such differences in our replication. Besides, the *in vitro* experiments were carried out independently at least three times. ASA were dissolved in DMSO, and 0.1% DMSO (v/v) as a final concentration was used as a vehicle control for all experiments *in vitro*.

### Culture of MC-3T3E1 Preosteoblast Cell Lines

The MC3T3-E1 preosteoblast cell line was purchased from ATCC (Manassas, VA, United States) and maintained in ɑ-MEM, 10% FBS, 1% penicillin/streptomycin in an incubator at 37°C with 5% CO_2_. The cells were cultured in full ɑ-MEM for proliferation and in full ɑ-MEM plus 10 mM β-glycerophosphate (β-GP) and 50 mg/ml ascorbic acid (AA) for osteoblastogenesis.

### CCK8 Assay

We evaluated the effect of ASA on BMMs cell proliferation using CCK-8 assay. Cells were seeded in 96-well plates at a density of 3 × 10^3^ cells/well in full medium with M-CSF (30 ng/ml) and incubated overnight to adhere. Then, the cells were cultured in medium with indicated concentrations of ASA in triplicate for different days. After that, the cells were incubated with CCK8 solution (10 μL/well) at 37°C for an additional 2 h. The optical density (OD) at a wavelength of 450 nm was quantitatively measured with an Infinite F200 PRO absorbance microplate reader (Tecan). Cell proliferation (%) was analyzed relative to the control.

### TRAP Staining

BMMs were seeded in 96-well plates at a density of 3 × 10^3^ cells/well and exposed to 30 ng/mL M-CSF and 50 ng/ml RANKL for 5-7 days to obtain mature OCs which were classified as multinucleated cells in tartrate-resistant acid phosphatase (TRAP) staining assay. For TRAP staining assay, multinucleated OCs from BMMs were fixed with 4% paraformaldehyde. Then we used a leukocyte acid phosphatase kit (Sigma) to visualize multinucleated OCs which was performed according to the manufacturer’s instructions. Cells were incubated at room temperature for 30 min. Images of multinucleated cells were obtained and quantified under an Olympus microscope. TRAP-positive cells in different divisions such as mononuclear, multinuclear (2–5 nuclei; 5-10 nuclei) and giant cells (>10 nuclei) were scored and then analyzed respectively. The average number of TRAP-positive in each division in the control group was defined as 100%.

### F-Actin Ring Staining

BMMs were seeded in 6-well plates at a density of 2 × 10^5^ cells/well and exposed to 30 ng/mL M-CSF and 50 ng/ml RANKL with or without ASA for 5-7 days to obtain mature OCs. For actin cytoskeleton staining, cells were fixed with 4% paraformaldehyde. Then we used iFluor™ 555-Phalloidin working solution to visualize multinucleated OCs which was performed according to the manufacturer’s instructions. Cells were incubated at room temperature for 30 min. Then cells were washed in PBS for three times and incubated with DAPI (Sigma-Aldrich) for 5 min. Images of multinucleated cells were acquired under a ZEISS fluorescence microscope.

### Bone Resorption Assay

The procedure of bone resorption assay was described previously (Suda et al., 1999; Binder et al., 2009; Liu et al., 2019). Flat bone slices at 100 μM thick from bovine femur were used in bone resorption assay. BMMs were seeded on bone slices in 96-well plates overnight. The next day BMMs were induced by M-CSF and RANKL for 5-6 days, followed by M-CSF, RANKL and ASA treatment for another 2 days. Bone slices were then fixed with 2.5% glutaraldehyde for 7 min, after which cells were removed with vigorous sonication in 0.25 M ammonium hydroxide for 3 times. The bone resorption lacunae or pits were then photographed using a confocal laser microscope with ×200 magnification since it was good enough to characterize the surface roughness of the bone slices (Boyde and Jones 1991). Pit area was analyzed by Image-Pro Plus. Three view fields of each bone slice were randomly selected for further analysis.

### RNA Extraction and Quantitative Real-Time PCR Analysis

qRT-PCR was performed using an ABI 7500 Sequencing detection system. Total mRNA was isolated using TRIzol reagent (Life Technologies, Carlsbad, CA) and then subjected to reverse transcription. Complementary DNA (cDNA) was obtained and used as a template targeting TRAP, CTSK, ATPasev0d2 (ATPase, H+ transporting, lysosomal v0 subunit d2), MMP-9, RANKL, ALP, OCN and β-actin using specific primers. All primer sequences were summarized below (Table 1). β-actin was used as an internal control. Indicated mRNA levels were normalized to β-actin mRNA.

**TABLE 1:** Sequences of all primers in quantitative RT-PCR analysis.

Gene	Primer sequence
TRAP	Forward 5’ - TCCCCAATGCCCCATTC - 3′
	Reverse 5’ - CGG​TTC​TGG​CGA​TCT​CTT​TG - 3′
Cathepsin K	Forward 5’ - GAA​GAA​GAC​TCA​CCA​GAA​GCA​G - 3′
	Reverse 5’ - TCC​AGG​TTA​TGG​GCA​GAG​ATT - 3′
ATP6v0d2	Forward5′-TTTGCCGCTGTGGACTATCTGC- 3′
	Reverse 5′-AGA​CGT​GGT​TTA​GGA​ATG​CAG​CTC- 3′
MMP9	Forward5′-GCTGACTACGATAAGGACGGCA- 3′
	Reverse 5′-TAG​TGG​TGC​AGG​CAG​AGT​AGG​A- 3′
RANKL	Forward 5′-AGC​CGA​GAC​TAC​GGC​AAG​TA-3′
	Reverse 5′-AAA​GTA​CAG​GAA​CAG​AGC​GAT​G-3′
ALP	Forward5′-TCATTCCCACGTTTTCACATTC-3′
	Reverse 5′-GTT​GTT​GTG​AGC​GTA​ATC​TAC​C-3′
OCN	Forward5′-GCCTTCATGTCCAAGCAGGA-3′
	Reverse 5′-GCG​CCG​GAG​TCT​GTT​CAC​TA-3′
β actin	Forward 5’ - CTG​TCC​CTG​TAT​GCC​TCT​G - 3′
	Reverse 5’ - ATGTCACGCACGATTTCC - 3′

### Protein Extraction and Western Blotting Analysis

Whole-cell lysates of BMMs were prepared in SDS lysis buffer, followed by incubation at 95°C for 5 min. Cell lysates were thus subjected to SDS-PAGE. Proteins were separated and transferred onto NC membranes by electroblotting, which were incubated with blocking buffer for 1 h. Then the membranes were incubated with targeted primary antibodies at 4°C overnight, washed three times with Tris‐buffered saline plus Tween (TBST) for 10 min each time, and incubated with secondary antibodies at room temperature for 2 h. Finally, the bands were detected via analysis of immunoreactivity using an Immobilon Western kit (MILLIPORE) and photographed by ImageQuant LAS 500 (GE Healthcare). The expressions of indicated proteins were qualified by Image J software.

### Oestrogen Deficiency Induced Osteoporosis Model

To study the effects of ASA on oestrogen deficiency induced bone loss in mice, female C57BL/6 mice at 8-weeks were ovariectomized (OVX) or subjected to a sham operation (SHAM) which was described previously (Liu et al., 2019). After recovery, SHAM-operated with vehicle treatment (group SHAM, control, *n* = 6), ovariectomized-operated with vehicle treatment (group OVX, control, *n* = 6), ovariectomized-operated with ASA treatment (group ASA, 30 mg/kg, *n* = 6) were administered intraperitoneally once a day. ASA was dissolved in the final concentration of 1.25% DMSO (v/v) and 2.5% Tween80 in saline (v/v) as a vehicle. The group SHAM and OVX mice also received a similar injection with 1.25% DMSO (v/v) and 2.5% Tween80 (v/v) in saline as control. The body weight was collected once a week since the body weight increased steadily after OVX operation as reported (Ghayor et al., 2015; Nishio et al., 2019). Animals were sacrificed after 4 weeks’ treatment. The femurs and tibias were dissected and fixed with 4% paraformaldehyde for 24 h for subsequent inspections, such as pQCT scanning, μCT scanning, TRAP staining and H&E staining. The livers, lungs, hearts, kidneys and spleens were also dissected and fixed with 4% paraformaldehyde for H&E staining.

### Micro-CT Scanning and Histomorphometric Analysis

Three-dimensional reconstructions of the tibias were obtained from images acquired using a high-resolution micro-computed tomography scanner (Skyscan 1172; Skyscan; Aartselaar, Belgium). The tibias image acquisition was carried out with same parameters as follows: pixel size, 10 μm; X-ray voltage, 50 kV; electric current, 500 μA; rotation step, 0.7°, in accordance with the recommendations of the American Society for Bone and Mineral Research (ASBMR) (Dempster et al., 2013). Then the scans were integrated into 3D voxel image and analyzed to quantitatively assess bone parameters using SkyScan CT Analyser software (version 1.15.4.0) according to standardized protocols.

Briefly, the proximal end of the tibias corresponding to 0–5 cm region below the growth plate was scanned. The images of the trabecular region of interest (ROI) extended from the 100th layer proximally to the end of the distal growth plate over 200 layers toward the diaphysis were contoured for trabecular bone analysis. Bone parameters such as bone volume/tissue volume (BV/TV), bone surface/tissue volume (BS/TV), bone surface/bone volume (BS/BV), trabecular bone number (Tb.N), trabecular thickness (Tb.Th) and the 3D images were obtained.

The femurs were decalcified in 10% EDTA for 3  weeks, paraffin-embedded, sectioned and stained by TRAP or HE solution. The histomorphometric examination was imaged using a Zeiss microscope with ×200 magnification. The percentage of the bone surface and osteoclast numbers were calculated according to standardized protocols.

### Ethical Use of Animals

C57BL/6 mice were purchased from SLAC laboratory and were maintained under standard animal housing conditions (55–60% humidity, 22-24°C with a 12/12-h light/dark cycle, free access to food and water). All animal experiments were conducted in accordance to the guidelines of the human use and care of laboratory animals and were approved by the Shanghai Jiao Tong University School of Medicine Animal Study Committee.

### Statistical Analysis

The results are expressed as the mean ± standard deviations. One-way ANOVA and two-tailed non-paired Student’s *t* test were used to compare differences, and statistical significance was displayed as *p < 0.05 **p < 0.01 or ***p < 0.001.

## Results

### α-Asarone Inhibits Murine Bone Marrow-Derived Macrophage Cells Osteoclastogenesis in a Dose- and Time-Dependent Manner

**FIGURE 1:**
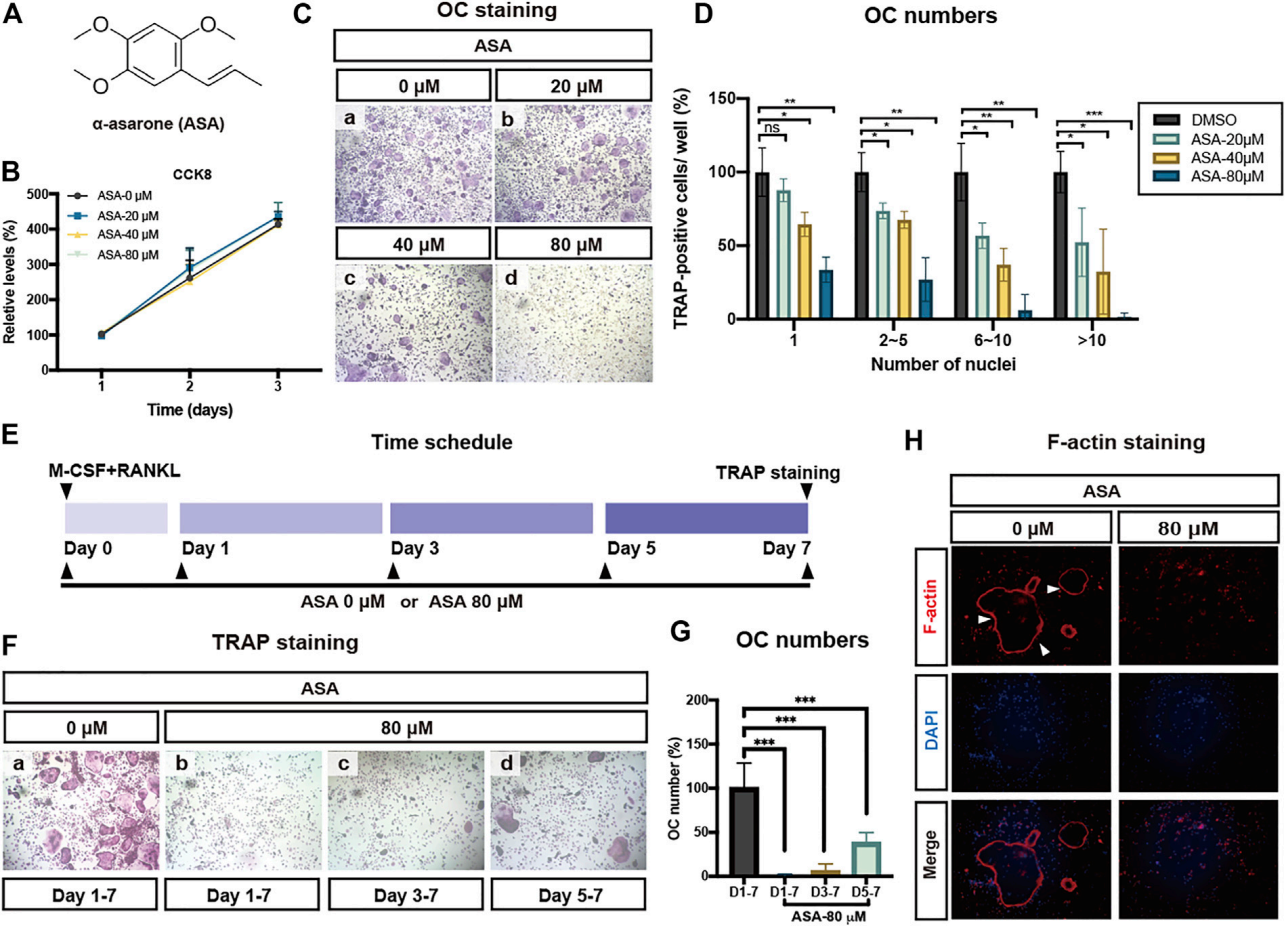
ASA inhibited OCs differentiation and actin structure formation without any cytotoxicity. **(A)** The chemical structure of ASA. **(B)** Effect of ASA on BMMs proliferation. Increasing doses of ASA (0.1% DMSO as a control) were incubated with BMMs which was sustained for proliferation by M-CSF for indicated days. Cell proliferation were determined by CCK8 assay. **(C)** Effect of ASA on OCs activation and differentiation. Indicated concentrations of ASA (0.1% DMSO as a control) were incubated with BMMs which were induced to differentiate into OCs by M-CSF and RANKL and then TRAP staining was applied to identify TRAP-positive multinucleated cells. **(D)** The numbers of TRAP-positive cells of different treatments as shown in **(C)** were calculated and presented graphically. The number of mononuclear, multinuclear (2–5 nuclei; 5-10 nuclei) and giant cells (>10 nuclei) was counted. The average number of TRAP-positive cells in each division in the control group was defined as 100%. The average numbers for the control group were 1207 for mononuclear cells, 152 for multinuclear cells (2–5 nuclei), 27 for multinuclear cells (5-10 nuclei) and 21.7 for giant cells (>10 nuclei). *p < 0.05, **p < 0.01, ***p < 0.001vs control. **(E)** Time schedule of experiments that determine the stage at which ASA blocked osteoclastogenesis **(F)** ASA inhibited RANKL-stimulated osteoclastogenesis at all stages, especially at early stage. **(G)** The numbers of TRAP-positive multinucleated cells of different treatments as shown in **(F)** were calculated and presented graphically. ***p < 0.001vs control. **(H)** BMMs were incubated with/without 80 μM ASA (0.1% DMSO as a control) for 5-7 days iFluorTM 555-Phalloidin (red) and DAPI (blue) staining was performed. Cells were observed and imaged using a ZEISS fluorescence microscope.

ASA structure was shown in Figure 1A. Murine BMMs were used to test the anti-osteoclastogenic activity of ASA. Two major events dominate the complex multi-step process of OCs formation: the proliferation of BMMs under the induction of M-CSF, and the subsequent differentiation of pre-OCs into mature OCs induced by RANKL. Prior to anti-osteoclastogenic activity, we investigate the effect of ASA in murine BMMs proliferation to find a safe dose *in vitro*. ASA in different doses were incubated with murine BMMs for different days, cell proliferation was then detected by CCK8 assay. As shown in Figure 1B, even at 80 μM, ASA did not exert any inhibitory effect in murine BMMs proliferation.

TRAP is an important and reliable indicator to identify OCs, therefor TRAP staining assay was applied to identify these osteoclast-like giant and TRAP-positive cells. BMMs were incubated with 50 ng/ml RANKL and 30 ng/mL M-CSF with different doses of ASA for 5-7 days. The TRAP staining results in Figure 1C indicated that ASA inhibited the formation of TRAP-positive multinucleated cells as well as mononuclear cells in a dose-dependent manner, which was confirmed by quantitative analysis of OCs numbers (Figure 1D) as well.

In Figure 1B, we have shown that even at 80 μM, ASA did not exert any inhibitory effect on M-CSF induced proliferation. To further determine which stage did ASA inhibit osteoclastogenesis, ASA (80 µM) was added to OCs differentiation medium beginning from different days shown in Figure 1E. The results showed that ASA could inhibit osteoclast differentiation at a time-dependent manner. ASA inhibited OCs osteoclastogenesis by 98.8, 94.4, 62.3% at early-stage, mid-stage and late-stage, respectively (Figure 1F and Figure 1G). Therefore, ASA was more efficient to prevent OCs osteoclastogenesis from early-stage (day1) and mid-stage (day3), rather than late-stage (day5).

F-Actin ring is a characteristic actin structure that is essential for mature OCs bone resorption. Hence, we further explored the effect of ASA on the formation of F-actin ring. The results showed pre-OCs could differentiate into mature OCs upon RANKL stimulation and cytoskeleton was then reorganized to create an F-actin-rich ring upon adhesion on the dish surface (Figure 1H). As shown in Figure 1H, the numbers of F-Actin ring were significantly decreased by 80 μM ASA, suggesting ASA has the potential to suppress actin-rings formation in OCs.

### α-Asarone Inhibited Osteoclasts Bone Resorption and Related Gene Expressions

**FIGURE 2:**
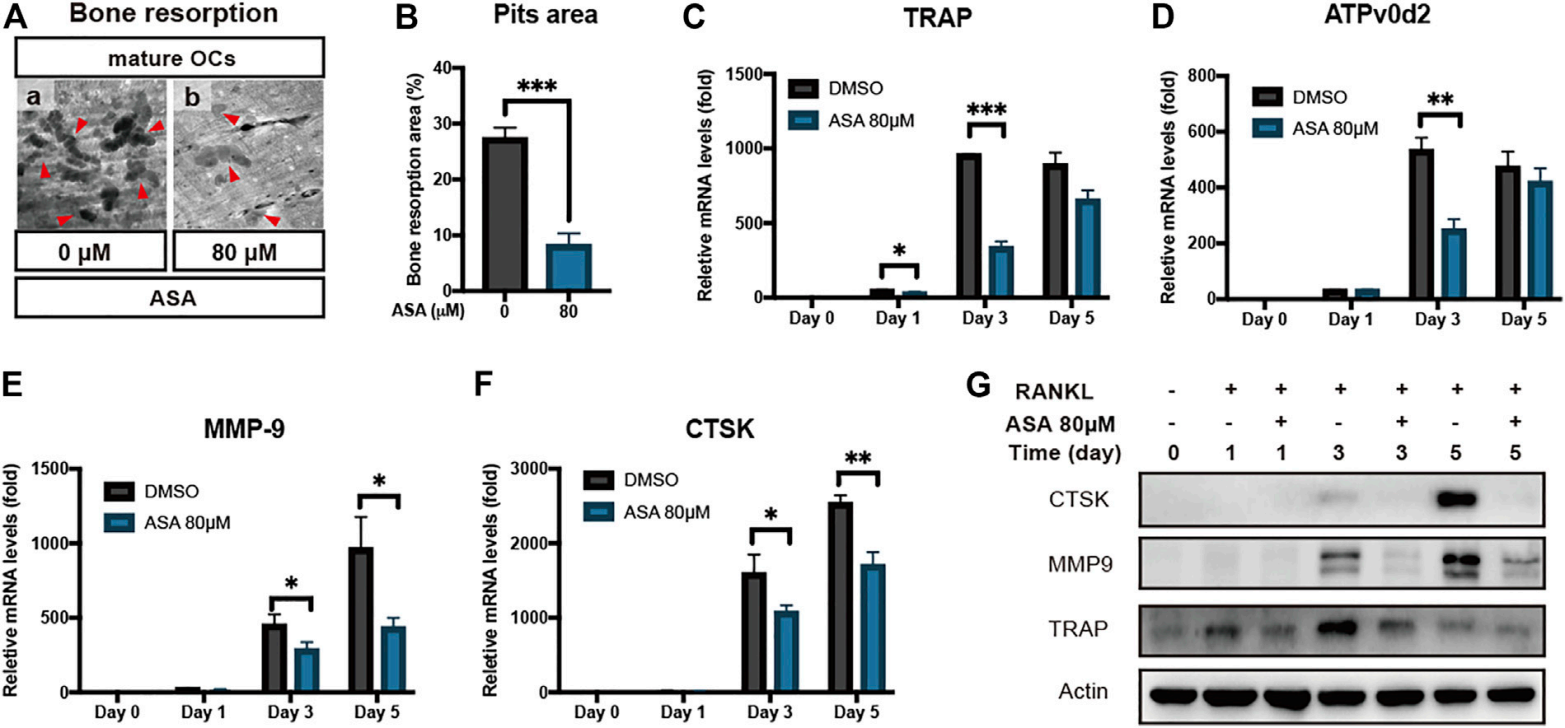
ASA inhibited RANKL-induced bone resorption and related gene expressions. **(A)** Effect of ASA on bone resorption of mature OCs. BMMs were plated on bone slices in 96-well plate and then differentiate into mature OCs by M-CSF and RANKL. Then ASA (0.1% DMSO as a control) was incubated with mature OCs for 2 days and bone slices were fixed. Images were shown as ×200magnification. The resorption pits in each image were indicated by the red triangles. **(B)** The pit areas were quantified using Image J software and presented graphically **(C)** BMMs were stimulated with M-CSF and RANKL in the presence of 80 μM ASA (0.1% DMSO as a control) for 1, 3, 5 days. OCs marker gene levels of **(C)** TRAP **(D)** ATPv0d2 **(E)** MMP9 **(F)** CSTK were determined by quantitative RT-PCR analysis. The results were normalized to β-actin expression and expressed as fold change relative to gene expression in control cells. *p < 0.05, **p < 0.01, ***p < 0.001 vs control **(G)** BMMs were stimulated with M-CSF and RANKL in the presence of 80 μM ASA (0.1% DMSO as a control) for indicated days. OCs marker protein level of CTSK, MMP9 and TRAP was detected by Western blotting. β-actin served as the internal control.

The formation of resorption pits on mineralized surface is a standard assay for assessing OCs function. Therefore, we detected the effects of ASA on the formation of bone resorption pits. Pre-OCs were placed on bone slices and were differentiated into mature OCs for 5-7 days, then ASA was added for two more days. The results in Figure 2A and the quantitative analysis in Figure 2B indicated that ASA (80 µM) reduced OCs bone resorption by 70.2%, compared with control group.

To further confirm the inhibitory effects of ASA on OCs differentiation and bone resorption, BMMs were induced into osteoclastogenesis and expressions of bone resorption-related gene in different stages were measured by RT-PCR and WB. As shown in Figures 2C–F, mRNA expressions of the bone resorption-related genes such as TRAP, ATPv0d2, MMP9 and CTSK were inhibited by ASA in different stages. We also detected the protein expression of CTSK, TRAP and MMP9, since they were largely responsible for cleaving and removing the bone matrix. As indicated in Figure 2G, protein level of CTSK, TRAP and MMP9 increased in a time-dependent manner upon RANKL stimulation, while ASA decreased its protein level, especially at day 5.

### α-Asarone Inhibited Osteoclasts Differentiation and Resorption Through AKT/p38/Iκb-α/p65 Followed NFATc1/c-Fos Pathway

**FIGURE 3:**
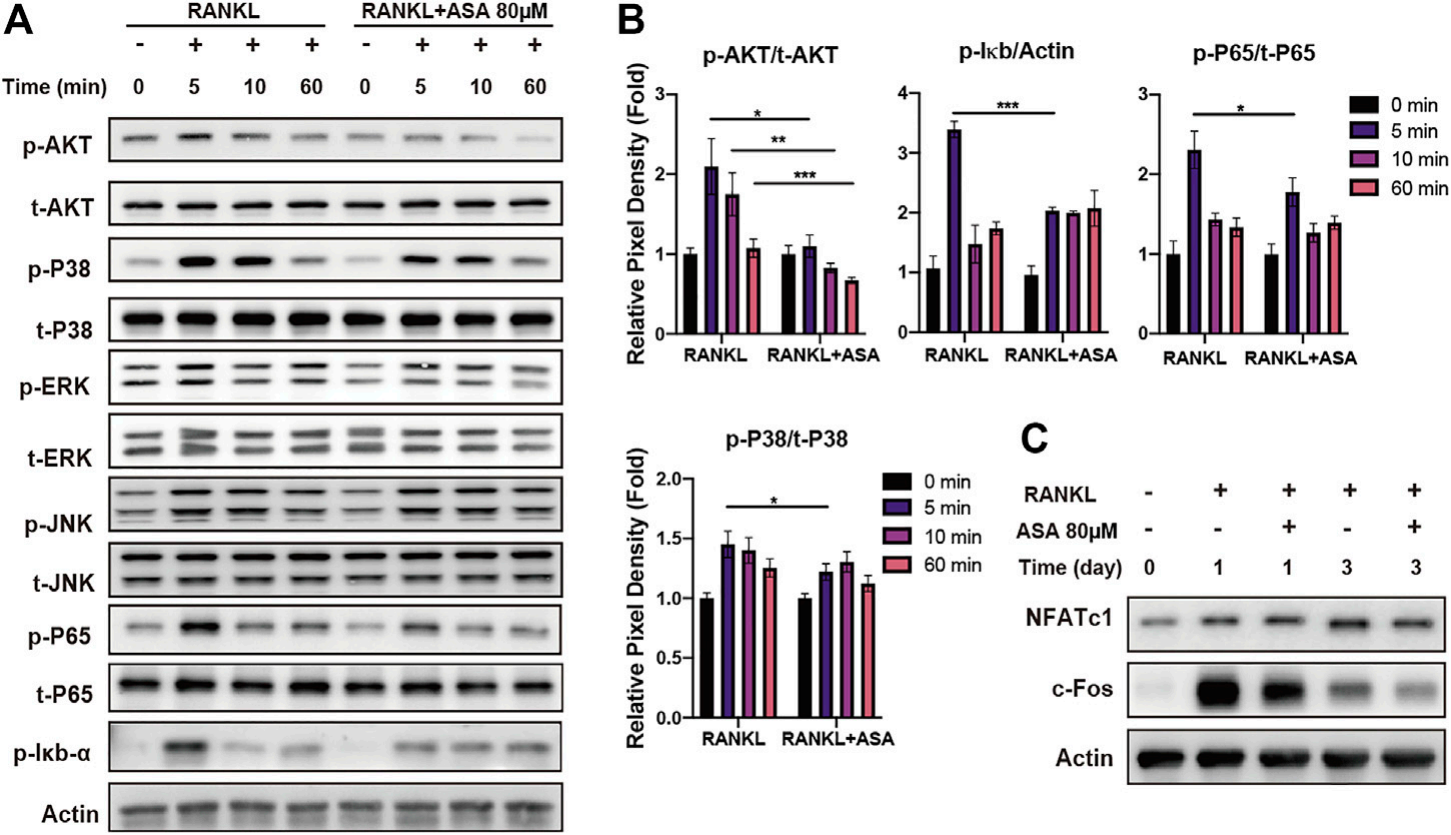
ASA attenuated RANKL-induced AKT, p38 phosphorylation and NF-κB signaling through NFATc1/c-fos. **(A)** BMMs were treated with/without 80 μM ASA (0.1% DMSO as a control) for 2 h, followed by 50 ng/ml RANKL for the indicated miniates. Cell lysates were then subjected to Western blotting analysis for p-AKT, t-ATK, p-p38, t-p38, p-IκB-α, p-p65, t-p65, p-ERK, t-ERK, p-JNK1/2, t-JNK1/2. **(B)** The quantified p-AKT/p-IκB-α/p-p65/p-p38 protein expression was normalized to total AKT/actin/p65/p38, relatively. *p < 0.05, **p < 0.01, ***p < 0.001 vs control **(C)** BMMs were treated by 50 ng/ml RANKL in the presence of ASA (0.1% DMSO as a control) for 1, 3, 5 days. Cell lysates were then subjected to Western blotting analysis for NFATC1, c-fos and β-actin. β-actin served as the internal control.

Phosphorylation of ERK1/2, JNK1/2, p38 and AKT are known to play a role in the early stage of RANKL induced OCs differentiation. As shown in Figures 3A,B, BMMs were treated by 50 ng/ml RANKL for indicated time, with or without pre-treatment 80 μM ASA for 2 h, then WB was applied to investigate phosphorylation of these signal molecules. The blots and quantification in Figures 3A,B indicated all signaling molecules were activated within 5-10 min, and peaked at 5 min, while the phosphorylation of AKT and p38 were attenuated upon ASA treatment. There was no difference in ERK1/2 and JNK1/2 phosphorylation. NF-κB signaling is also important for OCs differentiation. The phosphorylation IκB-α and p65 were activated by RANKL and then p65/p50 heterodimers would translocate to the nucleus to induce expressions of c-Fos and NFATc1. Thus, the phosphorylation of IκB-α and p65 in NF-κB signaling pathway were also detected. As show in Figures 3A,B, the phosphorylation of IκB-α was promoted by RANKL within 5 min subsequently by p65 phosphorylation, while p-IκB-α and p-p65 were both attenuated by ASA treatment.

NFATc1/c-fos, the most important transcription factors in osteoclastogenesis, controlled the expression of OCs differentiation and resorption related genes expressions such as TRAP, CTSK, MMP9, ATPv0d2 and so on. Thus, the inhibitory effect of ASA on the expression of c-Fos and NFATc1 was also evaluated by WB analysis. As shown in Figure 3C, RANKL induced the expression of c-Fos and NFATc1 both at the early-stage (day1) and mid-stage (day 3) of osteoclastogenesis. In particular, ASA strongly blocked RANKL-induced protein expression of c-fos at day1 and day3, with mild inhibitory effects on NFATc1 at day3.

All above results concluded that the anti-osteoclastogenesis effects of ASA in OCs differentiation and resorption were related with AKT, p38 phosphorylation as well as NF-κB signaling pathway, followed by NFATc1/c-fos signal pathway, then TRAP, CTSK, ATPv0d2 and MMP9 expressions.

### α-Asarone Reverses Ovariectomized-Induced Osteoporosis *in vivo*

**FIGURE 4:**
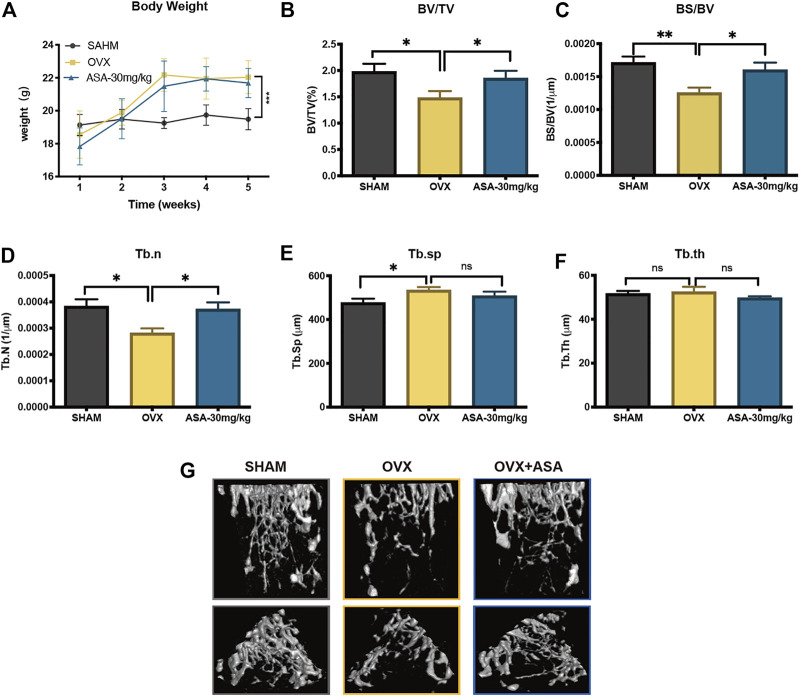
Therapeutic effects of ASA on oestrogen-deficiency OP *in vivo*. **(A)** The dynamics bodyweight changes of all group in 4-weeks ***p < 0.001 vs control mice. Tibias from all groups were analyzed by micro-CT. Bone parameters including **(B)** BV/TV **(D)** BS/BV **(E)** Tb. n **(F)** Tb. Sp **(H)** Tb. th of each sample were measured and calculated. *n* = 6. *p < 0.05 **p < 0.01, ***p < 0.001 vs control mice. **(G)** The 3D reconstructed images of tibias from each group are shown. The images represent tibias (upper panel, front view; lower panel, top view) of SHAM, OVX and ASA (30 mg/kg) treated mice after 4-weeks administration.

Oestrogen-deficiency is associated with bone loss and primary OP, resulting in an increased risk of fracture. We further investigated the effect of ASA on oestrogen-deficiency induced OP *in vivo*. OVX-operated mice were intraperitoneally administrated with 30 mg/kg ASA (1.25% DMSO+2.5% Tween80 in saline as vehicle control) for 4 weeks and then the tibias were isolated and scanned by microCT. As shown in Figure 4A, OVX-operated mice gained an average of 13% body weight during the study period, while ASA group did not show any significant change on body weight at week 4 compared to OVX-operated. As shown in the bone quality parameters analysis of ROI (Figures 4B–F), ASA increased BV/TV, BS/BV and Tb. n compared with OVX-group which indicated that ASA might improve the quality of bone microarchitecture. This protective effect was also indicated in 3D reconstructed images (Figure 4G), compared with SHAM operated mice, OVX-mice exhibited extensive OP while ASA improved it.

**FIGURE 5:**
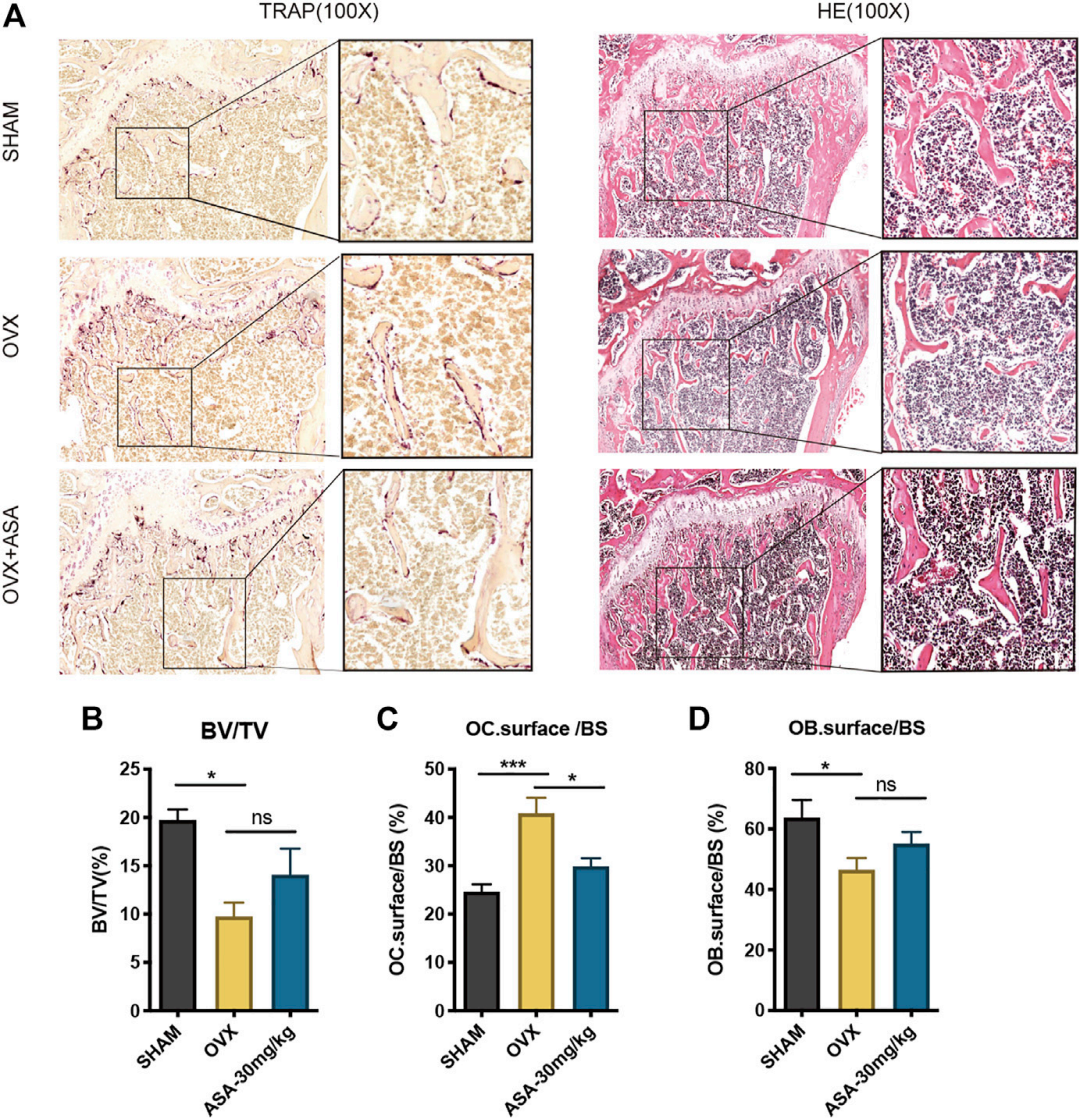
Effects of ASA OBs and OCs *in vivo*. **(A)** Mouse femurs were fixed, decalcified, dehydrated and sectioned. TRAP staining and H&E staining were applied to show OCs in different groups of SHAM, OVX and OVX + ASA (30 mg/kg). Regions that stained red are considered as TRAP-positive OCs. **(B)** The bone volume per field of bone tissue (BV/TV), the surface of OCs per bone surface (OC.surface/BS) and the surface of OBs per bone surface (OB.surface/BS) were analyzed.

In addition, the femurs were cut into slices and were subjected to TRAP and H&E staining (Figure 5A). The TRAP staining and statistics suggested that BV/TV were decreased by OVX and increased upon ASA treatment which were in accordance with microCT analysis (Figure 5B). Besides, OCs surface/BS *in vivo* were decreased upon ASA treatment, while OBs surface/BS were increased upon ASA treatment with no significance (Figures 5C,D).

## Discussion

OP is a chronic, metabolic and systemic skeletal disorder which may lead to numerous clinical and health-related consequences (Sözen et al., 2017; Compston et al., 2019). OCs play a prominent role in bone homeostasis. Therefore, modulation of OCs activity becomes an effective strategy against OP treatment. However, the clinically used anti-osteoclastognesis agents have some serious side effects, such as MRONJ (Kawahara et al., 2021). Thus, we determined to use the repurposing strategy to develop anti-osteoclastogenesis agents, since old drugs have gained sufficient safety data (Strittmatter 2014; Neuberger et al., 2018).

ASA is a naturally produced phenylpropene isolated from several plants especially in *Acorus tatarinowii*. Recently, lignin-like components purified from root tissues in *Acorus tatarinowi* have been reported to effectively suppress both RANKL induced osteoclastogenesis and bone resorption and thus aroused interest in the field of bone remodeling (Xu et al., 2016a; Zhang et al., 2018; Zhang et al., 2019). In order to illustrate the ASA effects on osteoclastogenesis *in vitro* and *in vivo*, in our present work, we investigated the efficacy of ASA on OCs proliferation and formation in primary murine BMMs during RANKL-induced osteoclastogenesis *in vitro*, as well as in the treatment of bone loss diseases *in vivo* as illustrated in an OVX-induced OP model.

Our results in Figure 1A showed that ASA inhibited RANKL-induced osteoclastogenesis without affecting BMMs proliferation from 20 to 80 μM. Evaluation of cell proliferation is essential for anti-osteoclastogenesis drug discovery to avoid the side effects on MRONJ, since the side effects of bisphosphonates may partially attribute to its decreasing effect on osteoclasts proliferation. In our present study, ASA exerted inhibitory effects on RANKL-induced osteoclast formation from 20 to 80 μM. A previous report suggested that ASA did not inhibit inhibitory effects at 10 μM (Zhang et al., 2019). This discrepancy may attribute to the different concentrations between the two studies. Besides, OCs function was depressed by ASA at 80 μM since the formation of OCs and F-actin ring was both disrupted. In Figure 1B, ASA at 80 μM almost totally inhibited the BMMs differentiation, while in Figures 2A,B, 80 μM ASA inhibited about 70.2% of mature OCs bone resorption. This discrepancy could be explained by the result in Figures 1E,F. It was shown that ASA not only exert inhibition of OCs differentiation at the early-stage, but also at the late-stage. That is why ASA inhibited mature OCs function by 70.2% which was in accordance to the inhibitory effects at the late-stage.

Many studies exploring the roles of MAPKs pathway in OCs metabolism have suggested that ERK, JNK, p38 as well as AKT are key players in RANKL-induced OCs differentiation and activation (Lee et al., 2018). Besides, RANKL also induces NF-κB signaling by recruiting TNF receptor-associated factor 6 (TRAF6) to RANK to activate a trimeric IκB kinase (IKK) complex. IKK complex induces phosphorylation and degradation of IκB-α to release of p65/p50 heterodimers. p65/p50 heterodimers would translocate to the nucleus to induce expressions of c-Fos and NFATc1, which are necessary for osteoclast precursor differentiation (Boyce et al., 2015). The results in Figure 3A indicated that AKT and p38 phosphorylation might participate in the inhibitory effect of ASA on osteoclastogenesis in BMMs. The phosphorylation of IκB-α was also attenuated upon ASA treatment. Then the decreased degradation of IκB-α held more p65/p50 heterodimers in an inactivate state in the cytoplasm, following by decreased expressions of NFATc1 and c-Fos (Figure 3C).

NFATc1 and c-Fos are the key regulators in osteoclastogenesis signal pathway. TRAP, an iron-containing enzyme was secreted into the resorptive vacuole beneath the ruffled border of the osteoclast to resorb bone matrix (van de Wijngaert and Burger 1986). CTSK is one of proteases active at osteoclast-resorptive compartments that plays a critical role in bone resorption, being largely responsible for cleaving and removing the organic bone matrix (Inaoka et al., 1995; Saftig et al., 1998). MMP9, a type IV collagenase is highly expressed in osteoclast cells and plays an important role in degradation of extracellular matrix (Wucherpfennig et al., 1994). ATPv0d2, a regulator of pre-osteoclast fusion, was an essential component of the osteoclast-specific proton pump that mediates extracellular acidification in bone resorption (Lee et al., 2006; Wu et al., 2009). Thus, our study illustrated that ASA decreased protein levels of NFATc1 and c-Fos (Figure 3B), subsequently followed by the lower expressions of various genes involving osteoclastic differentiation and function, such as TRAP, CTSK, ATPv0d2 and MMP-9 in a time-dependent manner (Figures 2C–F), which was in accordance to the decreased bone resorption (Figures 2A,B). To further confirm its potential on degradation of extracellular matrix, protein levels of CTSK, TRAP and MMP-9 were also detected. As indicated in Figure 2G, protein level of CTSK, TRAP and MMP9 increased in a time-dependent manner upon RANKL stimulation, while ASA decreased its protein level, especially at day 5.

OVX-induced rodent OP models, resembling the oestrogen deficiency context occurring in osteoporotic women, is the most widely employed model in the discovery of anti-resorptive agents (Stephens et al., 2021). Thus, the OVX-induced OP mouse model was employed to study the effect of ASA on bone destruction *in vivo*. ASA has been clinically used for the treatment of respiratory disorders and epilepsy at the dose of 3 mg/kg in adults (180 mg/day). It was estimated that the mice would need 27.3 mg/kg to obtain an equivalent dose in adults. As summarized (Shin et al., 2014), the pharmacologically active dose of ASA *in vivo* ranged from 1.7 mg/kg to 200 mg/kg, while the LD_50_ of ASA for mice in intra-abdominal was 310 mg/kg. Thus, ASA was administrated at 30 mg/kg (i.p.) once a day for 4 weeks to investigate its anti-OP effects in current study.

SHAM-operated and OVX-operated mice were administrated with 30 mg/kg ASA (i.p.) for 4 weeks. Then the tibias were isolated and firstly scanned by peripheral Quantitative Computed Tomography (pQCT) scans (XCTResearchSA). The result in Supplementary Figure S1 indicated that OVX-operated decreased bone mineral density (BMD) compared with SHAM-operated while ASA increased BMD in OVX-operated mice. ASA also increased BMD in SHAM-operated, but it did not show significant difference. Thus SHAM, OVX and OVX + ASA were further subjected to microCT scans and analysis. It is indicated that ASA substantially improved structural deterioration of bone tissue as shown on BV/TV, BS/BV and Tb. n in Figure 5A. OC. surface/BS and OB. surface/BS analysis in Figures 5C,D indicated that OVX increased OC. surface/BS and decreased OB. surface/BS. OC.surface/BS was decreased upon ASA treatment, while OB. surface/BS was also increased without significant difference. To inspect the osteoblastogenesis effects *in vitro*, ALP and OCN expressions were shown noticeably elevated in MC3T3-E1 osteoblastogenesis in a dose-dependent manner upon ASA treatment, especially at 80 μM **(** Supplementary Figure S2B–C **)**. As an important cytokine in osteoclastogenesis, RANKL expressions was also induced by ASA *in vitro* (Supplementary Figure S2A). However, OC. surface/BS were decreased *in vivo*. To explain this discrepancy, we applied OBs and OCs co-culture experiment. As illustrated by TRAP, MMP9 and CTSK expressions, osteoclastogenesis in BMMs was also depressed by ASA treatment in co-culture system (Supplementary Figure S3A–C). Therefore, we may conclude that ASA might strongly target RANKL downstream signaling pathway to depress osteoclastogenesis, although it induced RANKL expression in MC3T3-E1 osteoblast cell line.

We also determined the potential visceral tissue injury induced by ASA at 30 mg/kg (i.p.) for 4 weeks. Liver, kidney, lung, heart and spleen tissue in the control and ASA groups were all subjected to histopathological examination via H&E staining. As shown in Supplementary Figure S4, compared with control group, kidney, heart and spleen tissue in the ASA group had no obvious pathological changes. Upon ASA treatment, the alveolar wall was thickened in lung tissue. As to liver tissue, the vacuolization of hepatocyte cytoplasm and the number of binucleate cells increased by ASA. Also, the nuclei of hepatocytes became in different sizes. It is indicated that this effective dose (ASA 30 mg/kg for 4 weeks) by i. p. against OP might exert some toxicity in mice. The results were in accord with the potential toxicology of ASA *in vivo* which had been summarized by Ranjithkumar Chellian (Shin et al., 2014).

In conclusion, our work provided the evidence that ASA blocked RANKL-induced osteoclastogenesis and bone resorption through AKT, p38 and p65 followed by NFATc1/c-fos pathway *in vitro.* It also improved the bone structure in an OVX-induced OP *in vivo*. The current work helped us to understand the new effect of old drug ASA in osteoclastogenesis. ASA may find its potential as a lead compound to treat excessive OCs activity-induced bone loss diseases and more structure optimization is further needed to avoid the potential toxicity.

## Data Availability

The original contributions presented in the study are included in the article/Supplementary Material, further inquiries can be directed to the corresponding authors.
