# Supplementary material for: α-Asarone Attenuates Osteoclastogenesis and Prevents Against Oestrogen-Deficiency Induced Osteoporosis
Source: Front Pharmacol. 2022 Mar 18;13:780590. doi: 10.3389/fphar.2022.780590 (PMC8971932; doi:10.3389/fphar.2022.780590)
Supplement: Supplementary file 1 [file DataSheet1.pdf]

Supplementary figure legends

Fig.S1 ASA increased the BMD in OVX-operated mice

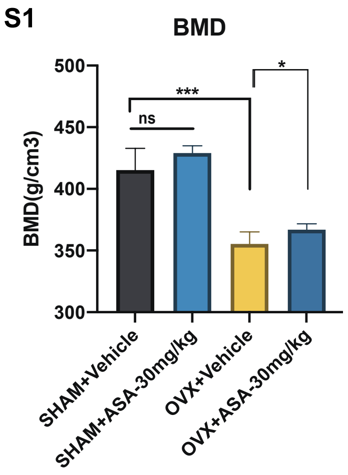

Tibias from all groups were analyzed by pQCT. bone mineral density (BMD) of each sample were measured and calculated. n=6. ns, no significance, *p<0.05 ***p<0.001 vs control mice.

Fig.S2 ASA increased MC3T3-E1 osteoblastogenesis.

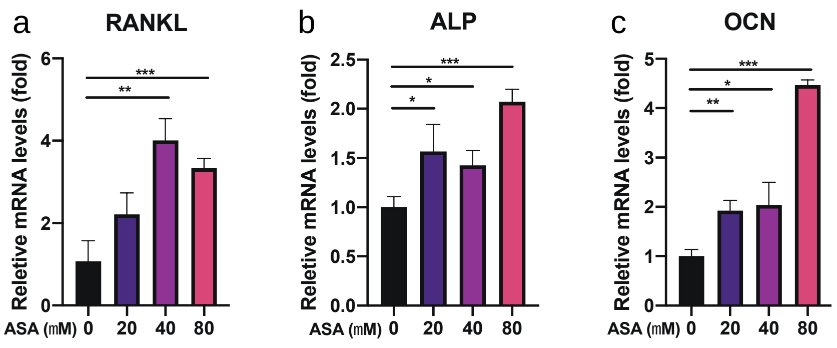

Effect of ASA on MC3T3-E1 osteoblastogenesis. Indicated concentrations of ASA were incubated with MC3T3-E1 differentiated into osteoblasts for 4 days and then mRNA level was detected. Osteoblastogenesis related gene levels of RANKL, ALP, OCN were determined by quantitative RT-PCR analysis. The results were normalized to β-actin expression and expressed as fold change relative to gene expression in control cells. *P<0.05, **P<0.01, ***P < 0.001 vs control.

Fig.S3 ASA decreased osteoclastogenesis in OBs and OCs co-culture experiment.

Effect of ASA on OBs and OCs co-culture experiment. MC3T3-E1 osteoblast cells were seeded in the hanging cell culture inserts (Millipore, MCHT12H48, 0.4μm). When MC3T3-E1 reached 90% confluence, the medium was changed into osteogenic medium (α-MEM supplemented with10% FBS, 1% penicillin–streptomycin solution, 10^{-8} M $VitD_3$ and 10^{-6} M PGE_2) with or without ASA (80μM) for 4 days. Meanwhile, BMMs were isolated and stimulated by M-CSF for 2 days. When co-culture, MC3T3-E1 in the hanging cell culture

inserts were co-cultured with BMMs for another 3 days. TRAP, MMP9 and CTSK gene expressions were then determined by quantitative RT-PCR analysis. The results were normalized to β-actin expression and expressed as fold change relative to gene expression in control cells. *P<0.05, **P<0.01, ***P < 0.001 vs control.

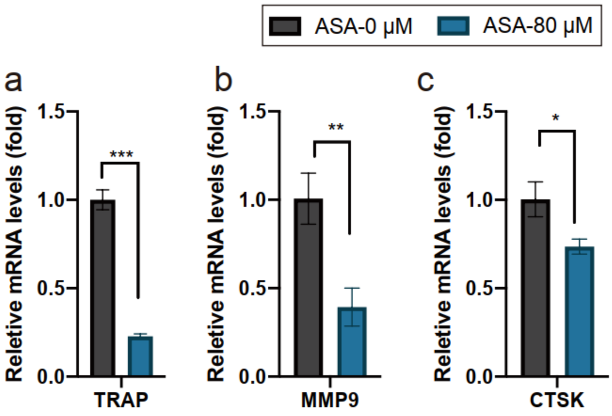

Fig.S4 ASA showed biotoxicity in livers and lungs

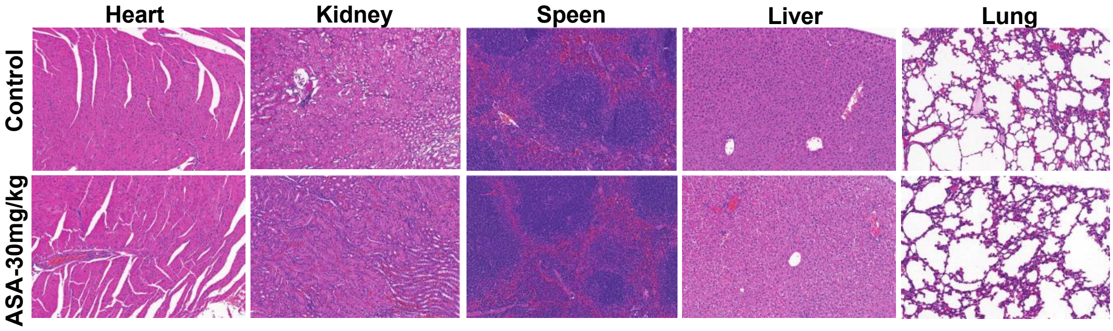

SHAM-operated C57BL/6J mice were i.p. injected with the vehicle (1.25% DMSO (v/v) and 2.5% Tween80 in saline (v/v)) and ASA (30mg/kg) for 4 weeks. Representative H&E images of heart, kidney, spleen, liver, and lung sections (n=4).
